# Evaluation of the Bone Reporting and Data System (Bone-RADS) on computed tomography in children with solitary bone lesions

**DOI:** 10.1007/s00247-026-06681-4

**Published:** 2026-06-09

**Authors:** Dilek Sağlam, Hasan Emin Kaya, Başak Erdemli Gürsel, Ülkünur Özdemir, Gökhan Gökalp, Bartu Sarısözen, Ulviye Yalçınkaya, Zeynep Yazıcı

**Affiliations:** 1 Deparment of Radiology, Uludag University Medical School, Gorukle, Nilüfer, Bursa, 16059 Turkey; 2 Department of Orthopedics, Bursa Uludağ University, Bursa, Turkey; 3 Department of Pathology, Bursa Uludağ University, Bursa, Turkey

**Keywords:** Bone-RADS, Bone tumors, Children, Computed tomography

## Abstract

**Background:**

The Bone Reporting and Data System (Bone-RADS) has been developed to facilitate and provide a common language for the management of bone tumors in adults. No bone reporting system has been developed to evaluate solitary bone lesions in children.

**Objective:**

This study aims to evaluate the interrater agreement and diagnostic performance of Bone-RADS in children with solitary bone lesions on computed tomography (CT).

**Materials and methods:**

From January 2014 to January 2026, patients <18 years old with bone lesions and a CT available in our picture archiving and communication system (PACS) were identified for eligibility. Five raters evaluated the CT images after a senior radiologist’s informative lecture on Bone-RADS. The final diagnosis, accepted as the reference standard, was determined by a multidisciplinary team based on biopsy or at least 2 years of follow-up. Interrater agreement was assessed. A binary Bone-RADS categorization was also used for diagnostic performance evaluation.

**Results:**

Seventy-four patients (22 females) with a mean age of 12.5±3.4 (range, 3-17) years old were enrolled in the study. The interrater agreement among all raters was almost perfect for both Bone-RADS and the binary Bone-RADS classification (*P*<0.001). Except for one non-advanced rater, all individual agreements with the reference standard were substantial in Bone-RADS (*P*<0.001). Binary Bone-RADS classification showed high accuracy, ranging from 81.1% to 90.5% across all raters.

**Conclusion:**

Bone-RADS on CT showed high interrater agreement and diagnostic performance in children with solitary bone lesions. Bone-RADS on CT may be used in children to manage solitary bone lesions.

**Graphical abstract:**

**Figure:**
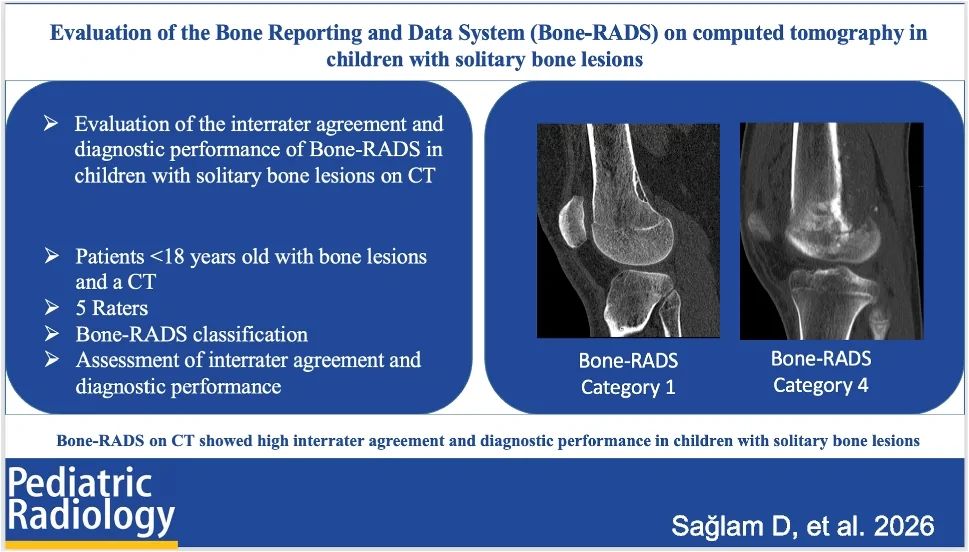

**Supplementary Information:**

The online version contains supplementary material available at https://doi.org/10.1007/s00247-026-06681-4.

## Introduction

Bone tumors comprise 4% of childhood cancers, and radiological investigation is one of the most important steps in the management of bone tumors [1]. The radiologist determines the level of suspicion for malignancy or the need for further evaluation. Radiological scoring systems, including Solitary Bone Tumor Imaging and Data System (BTI-RADS), Osseous Tumor Reporting and Data System (OT-RADS), Radiological Evaluation Score for Bone Tumors (REST), and Bone Reporting and Data System (Bone-RADS), have been developed to ease and provide a common language in the management of bone tumors [2–5]. All of these algorithms are developed for use in adults. No bone reporting system has been developed to evaluate solitary bone lesions in children.

Bone-RADS is developed by the Society of Skeletal Radiology. Computed tomography and MRI can both be used in Bone-RADS, as separate algorithms are available for each imaging modality [2]. This study aims to evaluate the interrater agreement and diagnostic performance of computed tomography (CT) in Bone-RADS in children with solitary bone lesions. To the best of our knowledge, this is the first study in the literature to evaluate solitary bone lesions with CT in Bone-RADS in children.

## Material and methods

### Study population

From January 2014 to January 2026, 251 patients with bone lesions were searched for eligibility. Inclusion criteria were <18 years old, presence of a solitary bone lesion, evaluation with CT available in our picture archiving and communication system (PACS), final diagnosis with biopsy, or at least 2 years of follow-up. Exclusion criteria were ≥18 years old, presence of multiple lesions, and absence of an available CT examination.

### Computed tomography evaluation

A senior radiologist gave an informative lecture before the raters’ evaluation of CT images. The lecture notes were derived from the study by Chang et al., which serves as the guideline for Bone-RADS on CT and magnetic resonance imaging (MRI) in adults [2]. This lecture lasted an hour and covered the Bone-RADS criteria, a detailed description of each morphological feature and the algorithm, and the pitfalls of specific lesions that should be carefully considered. The lecture content was also provided as a file to all raters for consultation if necessary during the evaluation process (supplementary). Bone-RADS categories were (1) likely benign; (2) incompletely assessed on imaging; (3) indeterminate; and (4) suspicious for malignancy or requiring treatment. Bone-RADS defines lesions as lytic, sclerotic, or mixed according to the density of the lesion. The lytic lesions and sclerotic or mixed density bone lesions have separate algorithms, leading to a category of Bone-RADS. In both algorithms, definitely benign lesions with typical imaging features, including fibrous dysplasia, non-ossifying fibroma, subchondral cyst, and macroscopic fat-containing lesions like intraosseous lipoma, and hemangioma, are Bone-RADS category 1. In each algorithm, the presence of concerning imaging features, which are cortical involvement, soft tissue extension, pathological fracture, or aggressive periosteal reaction, leads to Bone-RADS category 4. The lesions that cannot be categorized as Bone-RADS 1 or 4 belong to Bone-RADS categories 2 or 3, as determined by the algorithms. In the Bone-RADS evaluation, categories 2 and 3 were merged and assessed together because they may vary with the rater’s experience. The Bone-RADS algorithm includes clinical data such as pain attributable to the lesion and a history of malignancy. These were omitted in the present study, and a revised Bone-RADS algorithm without these data is used, as in the study by Haseli et al. [6]. Only morphological criteria for CT images, as expressed in the algorithm, were used.

Five raters, one pediatric radiologist (D.S.), two musculoskeletal radiologists (H.E.K., G.G.), one general radiologist (B.E.G.), and a 5th year radiology resident (U.O.), evaluated all CT images blinded to the final diagnosis and management of lesions. The advanced raters were D.S. (15 years of experience on pediatric radiology), H.E.K. (13 years of experience on musculoskeletal radiology), and G.G. (23 years of experience on musculoskeletal radiology), whereas the non-advanced raters were B.E.G. (14 years of experience on ultrasound) and U.O. (4 years of experience on general radiology). Raters were blinded to patients’ diagnoses and knew only patients’ age and sex. All lesions were categorized according to Bone-RADS by each rater.

The patients’ final diagnoses were accepted as the reference standard. Final diagnosis was determined by the multidisciplinary team, including a senior pediatric radiologist, a pediatric orthopedist, and a senior bone pathologist. The final diagnosis of lesions is based on either histopathological examination or on at least 2 years of follow-up.

A binary Bone-RADS categorization, with category 1 classified as negative and categories 2, 3, and 4 classified as positive, was also established to assess the diagnostic performance of Bone-RADS.

### Statistical analyses

Descriptive statistics and frequencies were given. Interrater reliability was assessed with Cohen’s kappa and Fleiss’ kappa. “Kappa values were interpreted as follows: <0 poor, 0.00–0.20 slight, 0.21–0.40 fair, 0.41–0.60 moderate, 0.61–0.80 substantial, and 0.81–1.00 almost perfect agreement.” Diagnostic performance was evaluated using accuracy, sensitivity, specificity, and positive and negative predictive values. Statistical analyses were conducted in R (R Core Team, 2022, Vienna, Austria) and IBM SPSS Statistics (version 29.0; IBM Corp, Armonk, NY, USA). *P*-values <0.05 were considered statistically significant.

## Results

Of the 251 patients, 76 had a CT available in our PACS. Two of them were excluded because of multiple lesions. Finally, 74 patients were enrolled in the study. Twenty-two of the patients were female. The mean age was 12.5±3.4 (range, 3–17) years old.

The most common localization of the lesions was the femur and tibia. The most common pathology was non-ossifying fibroma, followed by osteoid osteoma. Of 74 lesions, 19 (25.7%) underwent histopathological examination. The characteristics of the study population and lesions are given in Table 1.

**Table 1:** Characteristics of the study population

Characteristic	Number	Percentage	Value
Age (years)			
Mean – standard deviation			12.54-3.4
Minimum-maximum			3–17
Sex			
Female/male	22/52	29.7/70.3	
Localization			
Calcaneus	1	1.4	
Phalanx	4	5.4	
Femur	25	33.8	
Fibula	2	2.7	
Humerus	5	6.8	
Clavicle	1	1.4	
Phalanx	1	1.4	
Radius	4	5.4	
Scapula	4	5.4	
Talus	1	1.4	
Tibia	25	33.8	
Ulna	1	1.4	
Site			
Right/left	43/31	58.1/41.9	
Final diagnosis			
Aneurysmal bone cyst	2	2.7	
Atypical cartilage tumor	2	2.7	
Chondroblastoma	2	2.7	
Enchondroma	3	4.1	
Eosinophilic granuloma	1	1.4	
Ewing sarcoma	2	2.7	
Fibromyxoid proliferation	1	1.4	
Fibrous dysplasia	2	1.4	
Non-ossifying fibroma	18	24.3	
Osteoid osteoma	17	23.0	
Osteochondroma	9	12.2	
Osteomyelitis	3	4.1	
Osteosarcoma	6	8.1	
Simple bone cyst	5	6.8	
Supracondylar process	1	1.4	

**Fig. 1:**
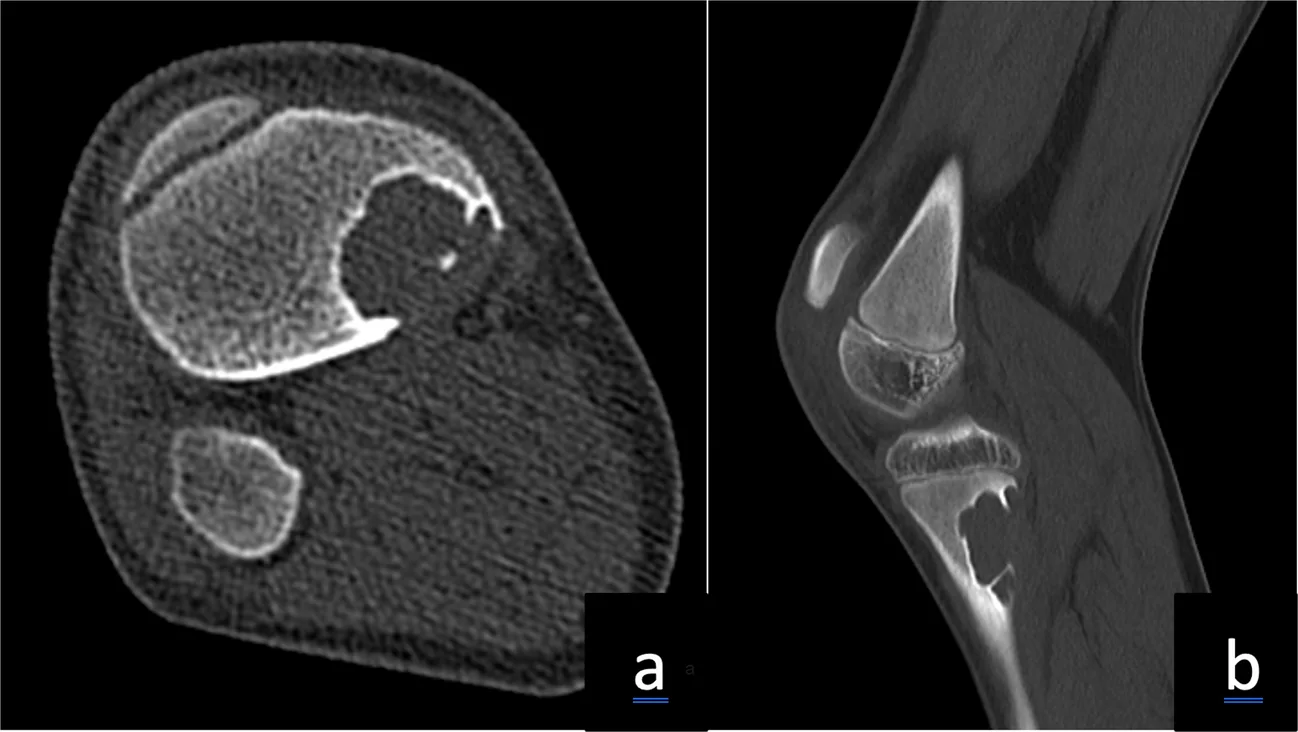
Axial (**a**) and coronal (**b**) computed tomography in bone window (WW 1500, WL 450) demonstrates a lytic lesion located in the proximal tibial metaphysis consistent with non-ossifying fibroma. Although definitely benign, the presence of cortical involvement makes the lesion Bone-RADS category 4. An advanced rater categorized it as Bone-RADS category 1

**Fig. 2:**
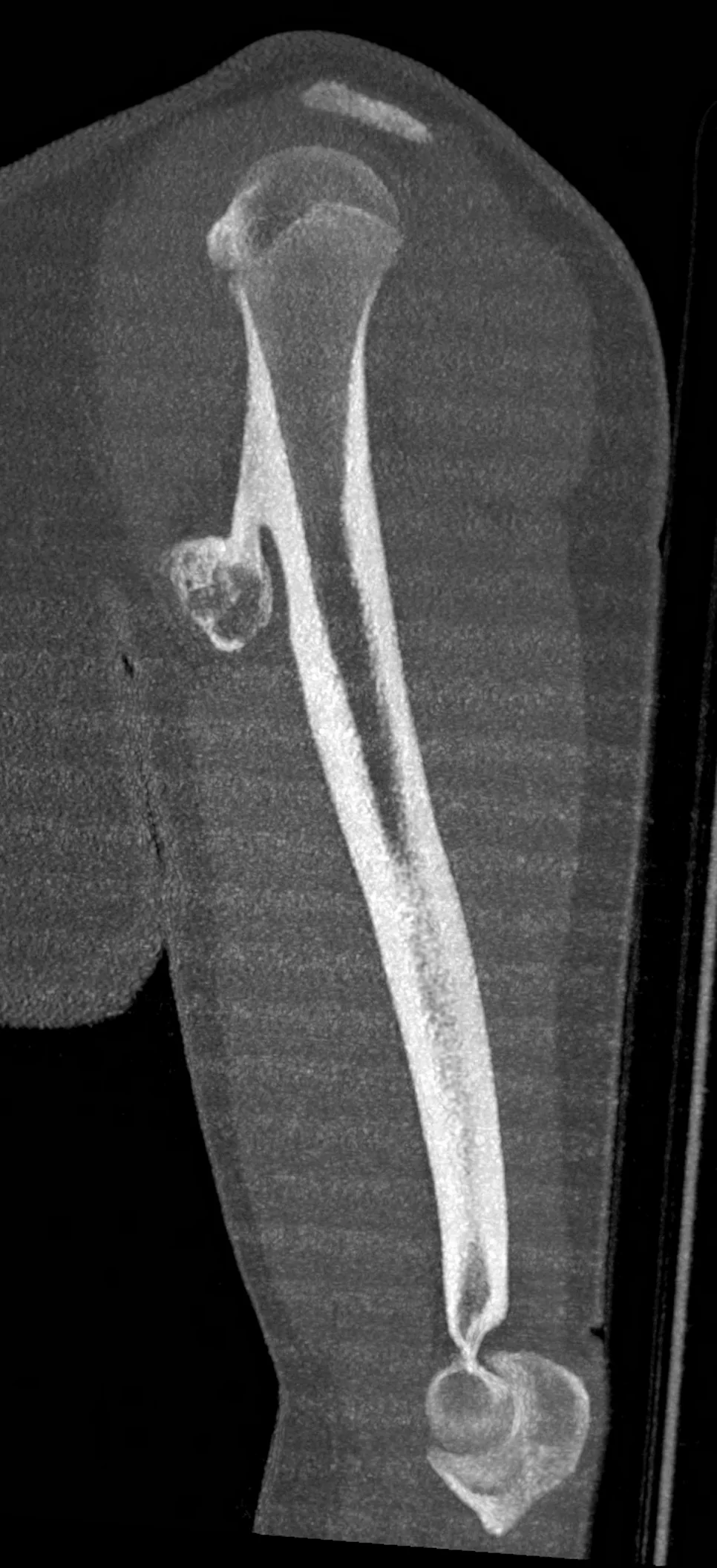
Coronal computed tomography in bone window (WW 1500, WL 450) shows an osteochondroma in the proximal humerus in a 16-year-old boy, which is definitely benign and Bone-RADS category 1. This was categorized as Bone-RADS category 3 by an advanced rater

The interrater agreement among all raters was almost perfect for both Bone-RADS and the binary Bone-RADS classification (Table 2). The interrater agreement among advanced raters was substantial for Bone-RADS and increased to almost perfect for the binary Bone-RADS classification. However, in non-advanced raters, both Bone-RADS and the binary Bone-RADS classification demonstrated moderate agreement. When individual agreement of raters was assessed, except for one non-advanced rater, all were substantial in Bone-RADS. Individual assessment of the binary Bone-RADS classification by all raters showed substantial to almost perfect agreement. The examples of false positive and false negative cases are presented in Fig. 1 and Fig. 2.

**Table 2:** Interrater agreements in Bone-RADS and binary Bone-RADS classification among raters

Rater	Analyses	Bone-RADS			Binary Bone-RADS classification		
		Kappa value	*P*-value	Degree of agreement	Kappa value	*P*-value	Degree of agreement
All raters	Fleiss	0.842 0.785–0.899	<0.001	Almost perfect	0.903 0.857–0.949	<0.001	Almost perfect
Advanced raters	Fleiss	0.795 0.705–0.885	<0.001	Substantial	0.866 0.791–0.940	<0.001	Almost perfect
Non-advanced	Cohen’s	0.493 0.318–0.668	<0.001	Moderate	0.528 0.334–0.722	<0.001	Moderate
Rater 1 vs RS	Cohen’s	0.749 0.608–0.889	<0.001	Substantial	0.749 0.608–0.889	<0.001	Substantial
Rater 2 vs RS	Cohen’s	0.749 0.603–0.895	<0.001	Substantial	0.776 0.633–0.919	<0.001	Substantial
Rater 3 vs RS	Cohen’s	0.676 0.526–0.826	<0.001	Substantial	0.662 0.494–0.830	<0.001	Substantial
Rater 4 vs RS	Cohen’s	0.781 0.657–0.905	<0.001	Substantial	0.806 0.670–0.941	<0.001	Almost perfect
Rater 5 vs RS	Cohen’s	0.582 0.415–0.748	<0.001	Moderate	0.617 0.437–0.798	<0.001	Substantial

*Bone-RADS*, Bone Reporting and Data System; *RS*, reference standard

Binary Bone-RADS classification showed high accuracy, ranging from 81.1 to 90.5 across all raters. Sensitivity and specificity across all raters ranged from 82.9% to 100% and from 66.7% to 81.8%, respectively. The diagnostic performance of raters is given in Table 3.

**Table 3:** Sensitivity, specificity, positive predictive value, negative predictive value, and accuracy of binary Bone-RADS classification

Rater	Sensitivity (estimate 95% CI)	Specificity (estimate 95% CI)	PPV (estimate 95% CI)	NPV (estimate 95% CI)	Accuracy (estimate 95% CI)
1	100 (91.4–100)	72.7 (54.5–86.7)	82 (72.3–88.8)	100 (85.7–100)	87.8 (78.2–94.3)
2	100 (91.4–100)	75.6 (57.7–88.9)	83.7 (73.7–90.6)	100 (86.3–100)	89.2 (79.8–95.2)
3	97.6 (87.1–99.9)	66.7 (48.2–82)	78.4 (69.1–85.5)	95.6 (75.7–99.4)	83.8 (73.4–91.3)
4	97.6 (87.1–99.9)	81.8 (64.5–93)	86.9 (76.3–93.2)	96.4 (79.4–99.4)	90.5 (81.5–96.1)
5	82.93 (67.9–92.8)	78.8 (61–91)	82.9 (71.3–90.5)	78.8 (64.9–88.2)	81.1 (70.3–89.2)

*PPV*, positive predictive value; *NPV*, negative predictive value; *CI*, confidence interval

## Discussion

This study demonstrated almost perfect agreement among overall raters in evaluating solitary bone lesions in children using Bone-RADS on CT. The studies performed in adults range in a wider spectrum; Xing et al. reported a lower level of interrater agreement, moderate, for Bone-RADS in evaluation of solitary bone tumors on CT in 328 adults [7]. Park et al. also reported interrater agreement from moderate to almost perfect in their study evaluating adults with solitary bone tumors using CT in Bone-RADS [8]. The high level of agreement in the present study is a promising result for the usage of Bone-RADS on CT in children.

From Bone-RADS to binary Bone-RADS classification, the interrater agreement within advanced raters was increased from substantial to almost perfect, while it stayed moderate within non-advanced raters. The interrater agreement among advanced raters was higher than among non-advanced raters, as expected. In the evaluation of the agreement of each rater with the reference standard, the interrater agreement among the advanced raters was all substantial in both Bone-RADS and binary Bone-RADS classification. So, each advanced rater’s performance was at an acceptable level. In Bone-RADS and the binary Bone-RADS classification, the interrater agreement of one of the non-advanced raters increased from moderate to substantial, and the other from substantial to almost perfect, respectively. This finding suggests that even non-advanced raters could classify lesions as either not requiring follow-up or requiring further work-up using the binary Bone-RADS classification. The higher level of agreement in the binary Bone-RADS classification compared to Bone-RADS among the overall raters indicates that Bone-RADS is reliable in differentiating lesions that warrant discontinuation of follow-up, its primary objective.

This study demonstrated high sensitivity and accuracy of the binary Bone-RADS classification on CT in children with solitary bone lesions. Xing et al. also reported high sensitivity (88.8% to 94.6%) and accuracy (78.0% to 86.6%) in the binary Bone-RADS classification of CT in adult solitary bone lesions [7]. However, the specificity in the present study (66.7–81.8%) was higher than the represented specificity in Xing et al.’s study (42.9% to 71.1%). As in these studies, Park et al. reported high sensitivity (68–95%) in their study evaluating adults with solitary bone tumors using CT in Bone-RADS [8]. However, in their study, specificity was low for lucent lesions (11%) and sclerotic/mixed lesions (27%). They also demonstrated moderate accuracy, ranging from 42% to 57%. Higher sensitivity across these studies may result from the wide range of pathologies that are classified as positive (categories 2, 3, and 4) by the algorithm in binary Bone-RADS classification. All lesions, including incomplete or indeterminate imaging (Bone-RADS categories 2 and 3), the need for treatment, or the presence of malignancy potential, are considered positive. So, only definitely benign or no-treatment lesions stay in the negative site. The characteristic CT imaging findings may have facilitated the categorization of the lesions. In the present study, non-ossifying fibroma was the most frequent lesion, with a Bone-RADS category 1, which may be one of the reasons for the high specificity. Xing et al. also reported that the study sample may have contributed to diagnostic performance across studies [7]. Secondly, in this study, the raters evaluated the entire imaging study, whereas in Xing et al.’s study, they evaluated a single image in a PowerPoint presentation. Thirdly, we used the revised Bone-RADS algorithm, which excludes clinical findings such as pain and a patient history of malignancy.

Bone-RADS is a scoring system used to evaluate solitary bone tumors and has separate algorithms for CT and MRI. Computed tomography provides morphological imaging similar to that of adults, except for unfused epiphyses, despite differences in MR imaging between children and adults, including red bone marrow and growth-related changes. We think this may be the reason for the high accuracy and interrater agreement in both overall raters and individual raters’ evaluation of solitary bone lesions in children using Bone-RADS on CT. Another reason for this result may be the unique role of CT in evaluating bone lesions, which better demonstrates their morphological features than MRI.

Radiography is the first-choice imaging modality and has a unique role in the evaluation of bone lesions. It gives clues about the presence of bone destruction, the matrix of the tumor, and the type of periosteal reaction, which guides the examiner about the aggressiveness of the lesion [9]. However, management of bone lesions requires advanced imaging, such as CT and/or MRI, and the Bone-RADS is used for incidental bone lesions detected on CT or MRI. In this study, we retrospectively included solitary bone lesions detected on CT in children. Some of these children had also been evaluated with radiographs or MRI. However, consistent with the objective of this study, which was to determine the usefulness of Bone-RADS on CT, only each subject’s CT examination was evaluated.

Our study has some limitations. First, the study sample was small because we used only CT images from our PACS and patients evaluated by the multidisciplinary team. Second, although 15 different pathologies were involved in this study, bone lesions in children remain a wider spectrum that may affect the performance of Bone-RADS. The number of malignant cases was limited. Also, this study evaluated both sclerotic and lucent lesions together rather than performing separate statistical analyses. Third, the informative lecture may not be sufficient for a non-musculoskeletal radiologist to become familiar with Bone-RADS, nor for a musculoskeletal radiologist. Finally, all the raters in the present study were radiologists. Including other specialists, such as orthopedists, as raters may offer a new perspective on the use of Bone-RADS. Because we think that, with the Bone-RADS algorithm as a guide, other specialties may also use Bone-RADS with appropriate training. This may provide a common language among subspecialties in the management of solitary bone lesions in children.

## Conclusion

In conclusion, Bone-RADS in CT revealed high interrater agreement and diagnostic performance in children with solitary bone lesions. These results showed that Bone-RADS may also be used in children to manage solitary bone lesions.

## Supplementary Information

Below is the link to the electronic supplementary material.

ESM1 (PDF 1.36 MB)

## Data Availability

No datasets were generated or analysed during the current study.
