# Supplementary material for: Evaluation of the Bone Reporting and Data System (Bone-RADS) on computed tomography in children with solitary bone lesions
Source: Pediatr Radiol. 2026 Jun 9;56(11):2520–6. doi: 10.1007/s00247-026-06681-4 (PMC13669122; doi:10.1007/s00247-026-06681-4)
Supplement: Supplementary file 1 [file 247_2026_6681_MOESM1_ESM.pdf]

BONE RADS COMPUTED TOMOGRAPHY SUPPLEMENTARY

BONE RADS CATEGORIES FOR INCIDENTAL BONE LESIONS

Category	Lesion definition	Recommendation
1	Likely benign	Leave alone
2	Incompletely assessed on imaging	Different imaging modality needed
3	Indeterminate	Follow-up imaging: 6, 6, and 12 mos
4	Suspicious for malignancy or need for treatment	Biopsy and/or referral to orthopedic oncology

CT classification of bone lesions: lucent, sclerotic, or mixed density.

DEFINITIONS

1) Lucent lesion

- Lower attenuation than normal trabecular bone → hypodense CT appearance
- > 90% of lesion volume should be lucent
- May contain solid non-sclerotic areas of soft tissue attenuation
    - Example: Giant cell tumor
- May contain blood products
    - Aneurysmal bone cyst

2) Sclerotic or mixed density lesion

<u>Sclerotic lesion</u>

- Higher density than the surrounding or adjacent trabecular bone.
- Sclerotic lesion: > 50% of the lesion has a higher density than the normal trabecular bone.
- Example: enostosis, sclerotic metastasis

<u>Mixed lesion</u>

- <50% of lesion is higher density than the normal trabecular bone.
- Combination of sclerotic and lucent areas (equivalent or near equivalent amount)
- Example: Fibro-osseous lesions, chondroid lesions, osteonecrosis, subchondral cysts

Note: If a lesion is not lucent according to the "lucent lesion" definition; consider it in the Sclerotic or mixed density lesion algorithm.

SOLİTARY LUCENT LESİON ON CT

- **Presence of concerning imaging features** → cortical involvement, soft tissue extension, pathologic fracture, or aggressive periosteal reaction) feature
  Cortical involvement → cortical tunneling, endosteal scalloping, bone remodelling-expansion, cortical thickening.
  Example: bone metastases, multiple myeloma, Langerhans cell histiocytosis, osteomyelitis.

- **Known malignancy** → FDG-PET, BONE SCAN, MRI (BONE RADS 2) or follow up (Bone RADS 3)

- **Macroscopic fat or fat determined using ROI (mean density < 10 HU)** → Bone RADS 1
  Example: Intra-osseous lipoma, hemangioma, and red marrow

- **Definitely benign lesions** → fibrous dysplasia, non-ossifying fibroma, enchondroma, subchondral cyst, and hemangioma → Bone RADS 1

- **Definitely benign, but involves concerning imaging features** → Bone RADS 4

- **Cortical lucent lesions** → osteoid osteoma, osteoblastoma,cortical metastasis

- **Medullary lucent lesions** → aneurysmal bone cyst, unicameral bone cyst, giant cell tumor, chondroblastoma, clear cell chondrosarcoma, myeloma, or metastasis

Solitary Lucent Bone Lesion on CT in Adults

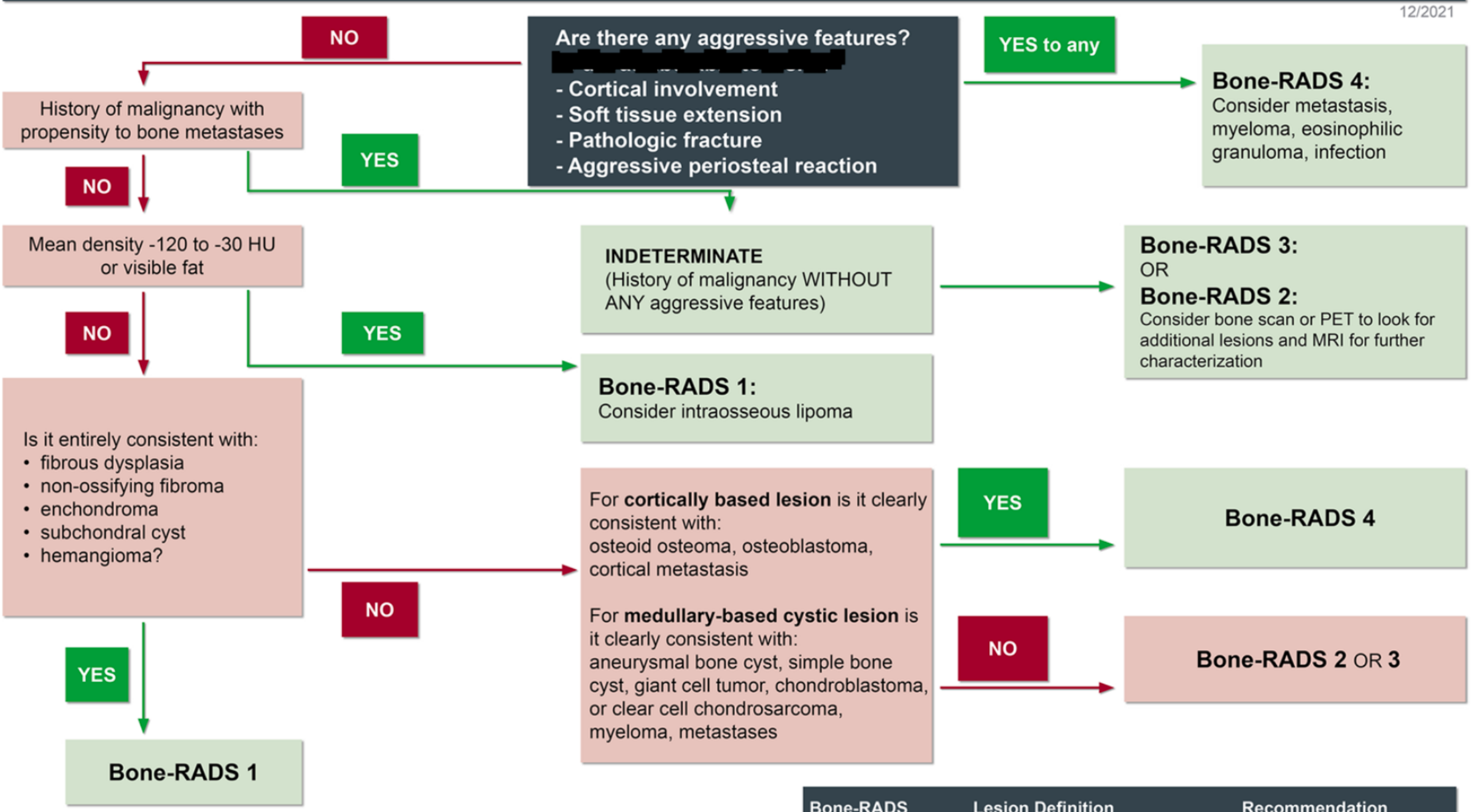

Bone-RADS	Lesion Definition	Recommendation
1	Likely benign	Leave alone
2	Incompletely assessed on imaging	Different imaging modality needed
3	Indeterminate	Follow-up imaging: 6, 6 and 12 months
4	Suspicious for malignancy or need for treatment	Refer to (orthopedic) oncology and consider biopsy

SOLİTARY SCLEROTİC/MİXED DENSİTY LESİON ON CT

- **Presence of concerning imaging features** → cortical involvement, soft tissue extension, pathologic fracture, or aggressive periosteal reaction) feature
  Cortical involvement → cortical tunneling, endosteal scalloping, bone remodelling-expansion, cortical thickening.
  Example: metastases, myeloma, chondrosarcoma, infection.

- **Known malignancy** → FDG-PET, BONE SCAN, MRI (BONE RADS 2) or follow up (Bone RADS 3)
- **Definitely benign lesions** → non-ossifying fibroma, enostosis, osteoma, sclerosing bone dysplasia → BONE RADS 1
- **Macroscopic fat or fat determined using ROI (mean density < 10 HU)** → Bone RADS 1

  Example: intraosseous lipoma, hemangioma, Paget disease, osteonecrosis, fibrous dysplasia, or non-ossifying fibroma (NOF).

- Ground glass → Fibrous dysplasia → BONE RADS 1
- Cartilage matrix
  - Concerning features present → BONE RADS 4
  - Concerning features not present → enchondroma → BONE RADS 1
- Enchondromas in axial skeleton → BONE RADS 4 (even not with aggressive features)
- Epiphyseal location → BONE RADS 4

Assessment of lesion density- ROI

Lesion that contain fat → often benign

Density measurement from non-contrast images

Endosteal scalloping for long bone

> 2/3 cortical thickness of extension of scalloping >2/3 craniocaudal lenght

Solitary Sclerotic or Mixed Density Bone Lesion on CT in Adults

02/2022

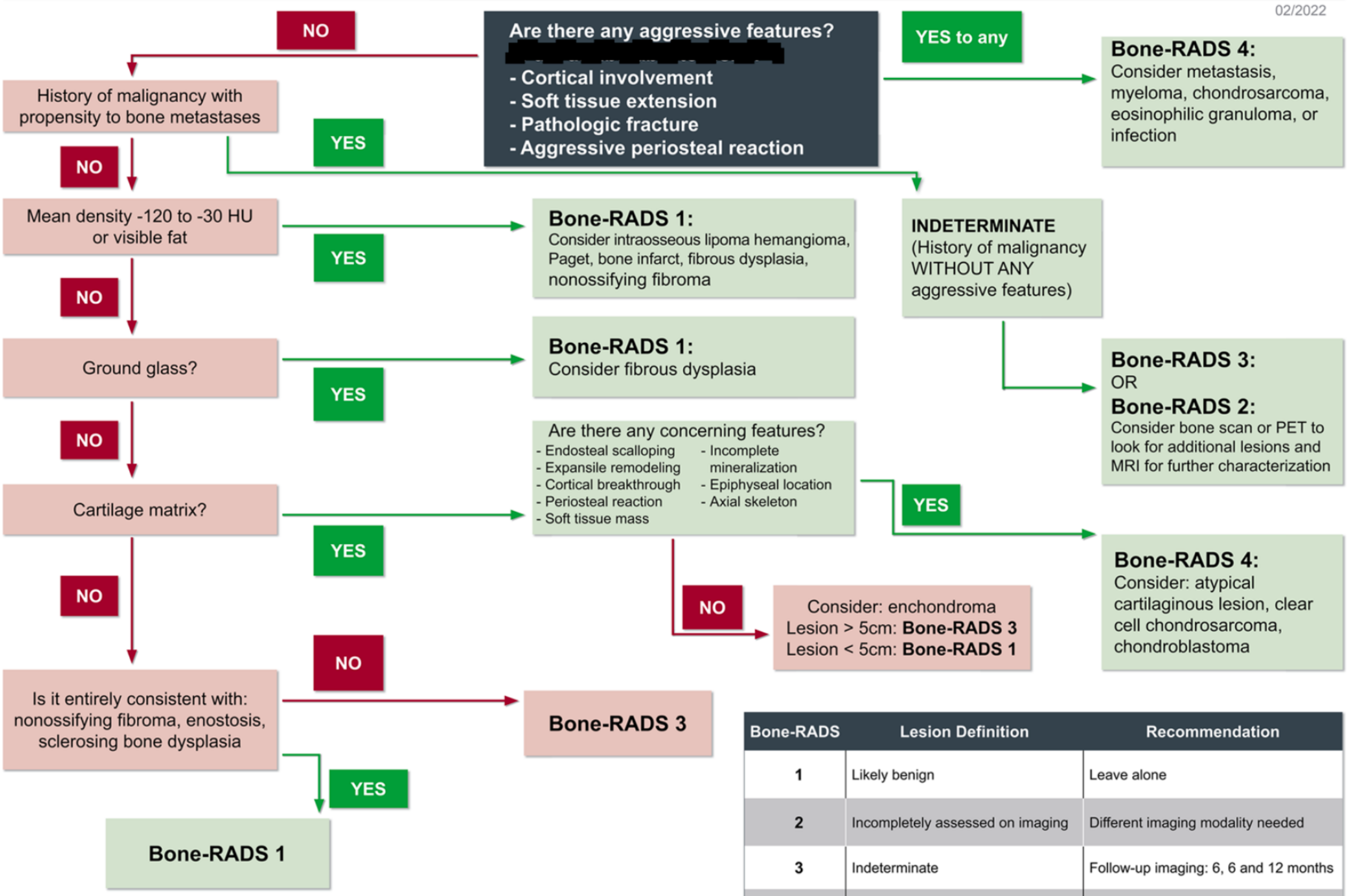

Bone-RADS	Lesion Definition	Recommendation
1	Likely benign	Leave alone
2	Incompletely assessed on imaging	Different imaging modality needed
3	Indeterminate	Follow-up imaging: 6, 6 and 12 months
4	Suspicious for malignancy or need for treatment	Refer to (orthopedic) oncology and consider biopsy
